# Genomic Characterization of *Haemophilus parasuis* SH0165, a Highly Virulent Strain of Serovar 5 Prevalent in China

**DOI:** 10.1371/journal.pone.0019631

**Published:** 2011-05-17

**Authors:** Zhuofei Xu, Min Yue, Rui Zhou, Qi Jin, Yang Fan, Weicheng Bei, Huanchun Chen

**Affiliations:** 1 State Key Laboratory of Agricultural Microbiology, Division of Animal Infectious Disease, College of Veterinary Medicine, Huazhong Agricultural University, Wuhan, China; 2 State Key Laboratory for Molecular Virology and Genetic Engineering, Institute of Pathogen Biology, Chinese Academy of Medical Sciences, Beijing, China; Naval Research Laboratory, United States of America

## Abstract

*Haemophilus parasuis* can be either a commensal bacterium of the porcine respiratory tract or an opportunistic pathogen causing Glässer's disease, a severe systemic disease that has led to significant economical losses in the pig industry worldwide. We determined the complete genomic sequence of *H. parasuis* SH0165, a highly virulent strain of serovar 5, which was isolated from a hog pen in North China. The single circular chromosome was 2,269,156 base pairs in length and contained 2,031 protein-coding genes. Together with the full spectrum of genes detected by the analysis of metabolic pathways, we confirmed that *H. parasuis* generates ATP *via* both fermentation and respiration, and possesses an intact TCA cycle for anabolism. In addition to possessing the complete pathway essential for the biosynthesis of heme, this pathogen was also found to be well-equipped with different iron acquisition systems, such as the TonB system and ABC-type transport complexes, to overcome iron limitation during infection and persistence. We identified a number of genes encoding potential virulence factors, such as type IV fimbriae and surface polysaccharides. Analysis of the genome confirmed that *H. parasuis* is naturally competent, as genes related to DNA uptake are present. A nine-mer DNA uptake signal sequence (ACAAGCGGT), identical to that found in *Actinobacillus pleuropneumoniae* and *Mannheimia haemolytica*, followed by similar downstream motifs, was identified in the SH0165 genome. Genomic and phylogenetic comparisons with other *Pasteurellaceae* species further indicated that *H. parasuis* was closely related to another swine pathogenic bacteria *A. pleuropneumoniae*. The comprehensive genetic analysis presented here provides a foundation for future research on the metabolism, natural competence and virulence of *H. parasuis*.

## Introduction

The Gram-negative bacterium *Haemophilus parasuis* is a strictly swine pathogen. It is a non-motile, pleomorphic NAD-dependent coccobacillus belonging to the family *Pasteurellaceae* of the γ-proteobacteria [1]. As a commensal colonizing the upper respiratory tract of pigs, *H. parasuis* is also an opportunistic etiologic agent that can cause serious systemic disease, namely Glässer's disease, which is characterized by fibrinous polyserositis, arthritis and meningitis, or acute pneumoniae and acute septicaemia [2]. In recent years, infections caused by this bacterial pathogen have led to great economic losses in the pig industry worldwide.

Based on the heat-stable antigens and the gel diffusion test, 15 serovars of *H. parasuis* have been classified, with apparent differences in virulence [3]. However, there are a large number of non-typeable *H. parasuis* strains exhibiting high heterogeneity at the molecular level [4], perhaps due to recombination or lateral gene transfer. *H. parasuis* serovar 4 predominates in North American herds, whereas serovar 5 and non-typeable strains are commonly isolated in many European countries [5], [6]. In China, the most prevalent serovars are 4, 5 and 13 [7].

To date, many potential virulence-associated factors investigated in the family *Pasteurellaceae* have been identified in *H. parasuis*, including lipopolysaccharide (LPS), capsular polysaccharide, fimbriae, outer membrane proteins, neuraminidase and iron acquisition systems [6], [8]–[10]. However, the molecular basis underlying these candidate virulence factors is yet to be fully elucidated.

Currently, the complete genome sequences of eight strains from four species within the genus *Haemophilus* are publicly available (up to June, 2010). These include *H. parasuis* SH0165 (GenBank accession no. CP001321), *H. ducreyi* 35000HP (AE017143), *H. influenzae* Rd KW20 (L42023), 86-028NP (CP000057), PittEE (CP000671), PittGG (CP000672), *Histophilus somni* 129PT (CP000436) and 2336 (CP000947). Among these, *H. influenzae* Rd and *H. somni* 129PT are both nonpathogenic strains of human and bovine origin, respectively, and *H. ducreyi* 35000HP causes chancroid, a human sexually transmitted disease [11], [12]. In this study, we carried out a comprehensive genomic characterization of *H. parasuis* SH0165, a high virulent serovar 5 strain recovered from the lung of a diseased piglet in North China. Comparisons of the genomic components between *H. parasuis* SH0165 and other representative species within the family *Pasteurellaceae* were performed in detail. Our work focused on identifying the particular genes/pathways that play a role in carbon metabolism, natural competence, virulence and host colonization of *H. parasuis*.

## Methods

### Bacterial strain and genome sequencing

*H. parasuis* strain SH0165 was isolated in 2001 from the lung of a diseased piglet in North China and was identified as serovar 5. This bacterial strain was cultured on tryptic soy agar (TSA) plates supplemented with 5% bovine serum and 10 µg/ml nicotinamide adenine dinucleotide (NAD). Bacterial DNA was extracted and sequenced using a shotgun sequencing strategy, as described previously [13].

### Sequence analysis

Glimmer3 [14], tRNASCAN-SE [15] and RNAmmer [16] were used to predict protein coding sequences (CDSs), tRNAs and rRNAs, respectively. BLASTP [17] was used for automated annotation based on sequence similarity in conjunction with searching the CDS set against the cluster of orthologous groups (COG) database, Kyoto Encyclopedia of Genes and Genomes (KEGG) database, Pfam protein families database and NCBI non-redundant protein database. A genome comparative circular map was implemented using the program CGview [18]. The sequenced draft genome of *H. parasuis* serovar 5 strain 29755 (NZ_ABKM00000000) was aligned to the complete genome of *H. parasuis* strain SH0165 (CP001321) using BLASTN (minimum sequence identity of 95% and expected threshold of 1e^−5^). Short DNA repeats in the genome sequences were searched with a Perl script *repeat_finder* (http://folk.uio.no/stephanf/repeat_finder.html) and graphical representations of the patterns in sequence conservation were created using WebLogo [19]. Protein transmembrane helices were predicted by the package TMHMM 2.0 [20].

### Ortholog identification and phylogenetic reconstruction

All protein sequences from the complete genomes of *H. somni* 129PT (CP000436), *H. influenzae* Rd (L42023), *H. ducreyi* 35000HP (AE017143), *Actinobacillus pleuropneumoniae* JL03 (CP000687), *A. succinogenes* 130Z (CP000746), *Aggregatibacter aphrophilus* NJ8700 (CP001607), *A. actinomycetemcomitans* D11S-1 (CP001733), *Pasteurella multocida* Pm70 (AE004439) and *Mannheimia succiniciproducens* MBEL55E (AE016827), were retrieved from the GenBank database. Protein sequences from the draft genome assemblies of *H. parasuis* strain 29755 were also retrieved. In this study, orthologous pairs of proteins between the CDS sets of two genomes were defined if protein identity was above 45%, alignment coverage above 70% and E_value below 1e^−20^. Orthologs present in the complete genomes of ten *Pasteurellaceae* species were retrieved. These protein sequences were aligned using the program MUSCLE 3.6 [21] and 727 alignments were then concatenated into a large alignment of 245,554 amino acids. A neighbor joining tree was reconstructed in the software MEGA 4 [22] *via* the bootstrap test of 1,000 replicates.

## Results and Discussion

### Genome structure and general features

**Figure 1:**
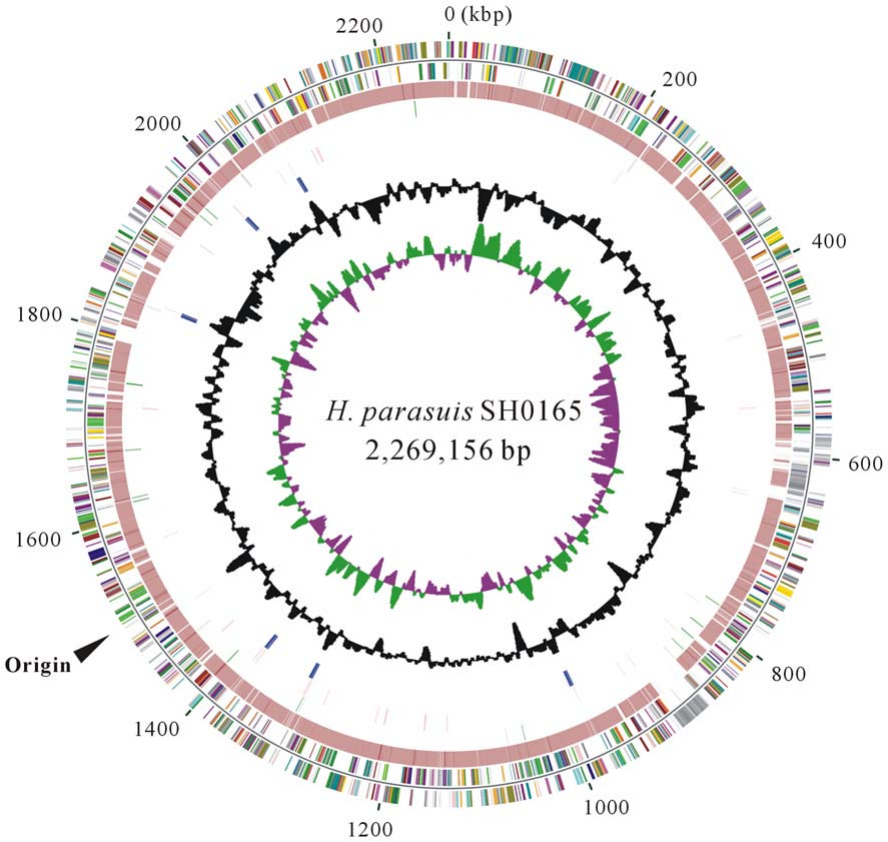
The circular representation of the complete genome of *H. parasuis* SH0165. Circles range from 1 (the outermost circle) to 8 (the innermost circle). The outer two circles show protein-coding genes on the forward and reverse strands in SH1605, colored according to COG categories. All genes are colored based on biological functions: maroon for translation, ribosomal structure and biogenesis; tomato for RNA processing and modification; navy for transcription; purple for DNA replication, recombination and repair; light brown for cell division and chromosome partitioning; gold for defense mechanisms; light purple for signal transduction; teal for cell envelope biogenesis and outer membrane; chocolate for intracellular trafficking, secretion and vesicular transport; aqua for posttranslational modification, protein turnover and chaperones; olive for energy production and conversion; lime for carbohydrate transport and metabolism; green for amino acid transport and metabolism; fuchsia for nucleotide transport and metabolism; light pink for coenzyme metabolism; red for lipid metabolism; orange for inorganic ion transport and metabolism; yellow for secondary metabolites biosynthesis, transport and catabolism; gray for general functional prediction; silver for function-unassigned conserved proteins; and black for unknown proteins not in the COG collection. The third circle shows the coordinates of BLAST hits of the *H. parasuis* SH1605 complete genome against genomic contigs of *H. parasuis* strain 29755 and these are colored in maroon. Fourth circle, insertion sequence elements in green. Fifth circle, tRNA genes in red. Sixth circle, rRNA operons in blue. Seventh circle, G+C content. Eighth circle, GC skew plot [(G−C)/(G+C); green indicates values >0; purple indicates values <0].

*H. parasuis* SH0165 contains a single, circular chromosome that is 2,269,156 base pairs (bp) in length (Figure 1). The chromosome encodes 2,031 protein-coding genes (192 pseudogenes not included herein), 56 tRNA genes and 20 rRNA genes on six ribosomal rRNA operons. Global characterizations of the *H. parasuis* SH0165 genome were summarized and compared to those of strains 129Pt of *H. somni*, Rd of *H. influenzae*, 35000HP of *H. ducreyi*, JL03 of *A. pleuropneumoniae* and Pm70 of *P. multocida* (Table 1). In comparison with the other *Haemophilus* spp., *H. parasuis* has a considerably larger chromosome, which is almost equal in size to those of *P. multocida* and *A. pleuropneumoniae*. The overall G+C content of the *H. parasuis* SH0165 genome was 40.0%, a bit higher than those of the other *Haemophilus* genomes. Pairwise nucleotide sequence alignments between the complete genome of *H. parasuis* SH0165 and the contigs of *H. parasuis* serovar 5 strain 29755 indicated that approximately 2,003 kb of sequence (above 95% identity) was shared by both strains, accounting for 88% of the total length of the SH0165 genome. There were 1,714 orthologous pairs of protein-coding genes between both *H. parasuis* strains of serovar 5, and *H. parasuis* strain SH0165 has 317 genes absent in strain 29755. It is worth noting that one third (104 genes) of these unique genes were phage-related. The *H. parasuis* SH0165 genome had about 198 CDSs encoding proteins involved in phage functions or phage islands, accounting for approximately 10% of the total CDSs. The majority of phage-related genes were mainly located in three phage islands designated HP-P1 (68.4 kb ranging from HAPS0536 to HAPS0638), HP-P2 (38.8 kb, HAPS0859–0910) and HP-P3 (9 kb, HAPS1147–1160). It has been suggested that the integration of phage elements, as a strategy of horizontal gene transfer, play a potentially important role in genetic diversity and virulence variations in many bacteria [23]. The phage-related genes found in the *H. parasuis* SH0165 genome may be also a putative contributor to virulence and inheritance differences.

**Table 1:** General features of whole genomes of *H. parasuis* (SH0165), *H. somni* (129Pt), *H. influenzae* (Rd), *H. ducreyi* (35000HP), *A. pleuropneumoniae* (JL03) and *P. multocida* (Pm70).

GenBank accession No.	CP001321	CP000436	L42023	AE017143	CP000687	AE017143
Strain	SH0165	129Pt	Rd	35000HP	JL03	Pm70
Total length (bp)	2,269,156	2,007,700	1,830,138	1,698,955	2,242,062	2,257,487
Number of CDSs	2,031	1,792	1,709	1,717	2,036	2,014
Average length of CDS (bp)	907	989	918	842	944	997
CDS genome coverage	81%	88%	86%	85%	86%	89%
GC content	40.0%	37.2%	38.2%	38.2%	41.2%	40.4%
Ribosome RNA						
16S rRNA	6	5	6	6	6	6
23S rRNA	6	5	6	6	6	6
5S rRNA	8	5	6	7	7	6
Number of tRNA	56	50	54	45	63	57

**Figure 2:**
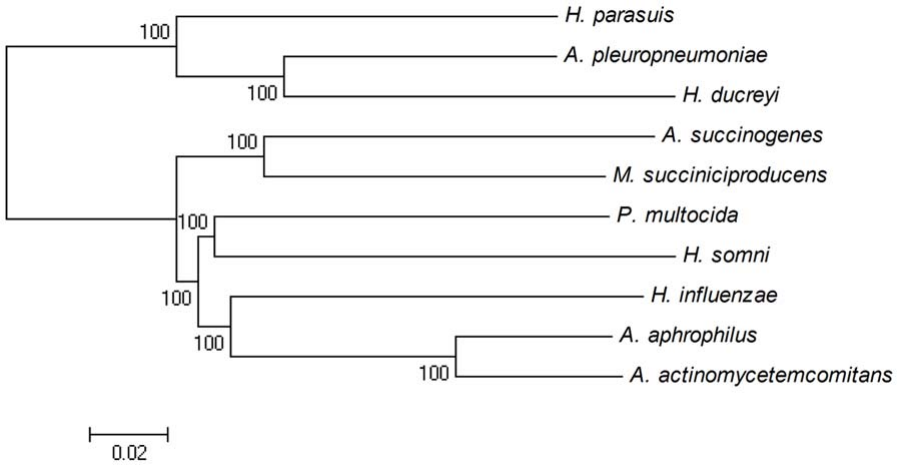
Neighbor-joining phylogeny. Tree derived from 727 concatenated, conserved protein sequences in the complete genomes of 10 *Pasteurellaceae* species. The scale indicates the number of substitutions per residue. Node support after 1,000 bootstrap replicates is shown.

Genome-wide comparisons of orthologous gene pairs between *H. parasuis* and other organisms within the family *Pasteurellaceae* showed that *H. parasuis* is more closely related to *A. pleuropneumoniae* (1,341 pairs of orthologs), which also colonizes the upper respiratory tract of pigs [24], but is more distantly related to the other three *Haemophilus* species, with only 1,095 orthologs found in *H. influenzae* (Table 2). Phylogenetic tree reconstruction based upon 727 conserved coding genes demonstrated that *H. parasuis*, *A. pleuropneumoniae*, and *H. ducreyi* were grouped into a subclade (Figure 2). However, the evolutionary relationship between *H. parasuis* and the strict human pathogen *H. ducreyi* is less obvious [25], as only 1,096 orthologs were identified in the genomes of both species. This may be due to adaptation of the two pathogens to different host species and tissues. These findings may indicate that, phylogenetically, the swine pathogens *H. parasuis* and *A. pleuropneumoniae* probably derive from a recent common ancestor and their adaptations reflect uniform host niches.

**Table 2:** Orthologs of predicted CDSs of *H. parasuis* SH0165 compared with complete genomes of related organisms.

	number of orthologs	% of CDSs in *H. parasuis*
Homologous to *A. pleuropneumoniae* JL03	1,341	66.0%
Homologous to *P. multocida* Pm70	1,213	59.7%
Homologous to *M. succiniciproducens* MBEL55E	1,184	58.3%
Homologous to *A. aphrophilus* NJ8700	1,149	56.6%
Homologous to *A. succinogenes* 130Z	1,145	56.4%
Homologous to *A. actinomycetemcomitans* D11S-1	1,111	54.7%
Homologous to *H. somni* 129PT	1,103	54.3%
Homologous to *H. ducreyi* 35000HP	1,096	54.0%
Homologous to *H. influenzae* Rd	1,095	53.9%

### Carbon source utilization

**Figure 3:**
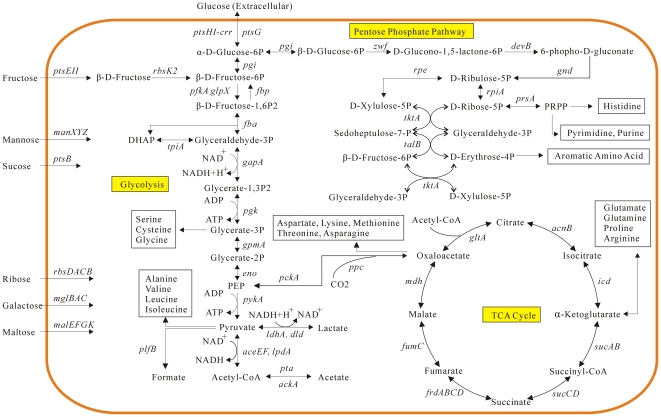
The metabolic pathways of central carbon flow in *H. parasuis* SH0165. Genes encoding key enzymes and functional proteins involved in the metabolic pathways are illustrated while the corresponding coding sequences (CDSs) indicated by HAPS numbers are list in Table S1 in the supplemental material.

*H. parasuis* is a facultative anaerobe which possesses metabolic pathways of both fermentation and respiration for energy generation [26]. Two kinds of sugar transport systems were identified in the *H. parasuis* SH0165 genome. We identified a predicted set of genes encoding ATP-binding cassette (ABC) transport complexes involved in the utilization of distinct sugars, including ribose (*rbsDACB*, HAPS1629–1632; *rbsACB*, HAPS1727–1725), galactose (*mglBAC*, HAPS0442–0444) and maltose (*malEFGK*, HAPS0236–0234, HAPS0237) (Figure 3). *H. parasuis* SH0165 also possesses genes encoding phosphotransferase systems (PTS), which can be devoted to the uptake of other sugars, including glucose (*ptsHI-crr*, HAPS0960–0958; *ptsG*, HAPS2092), fructose (*ptsEII*, HAPS0193–0189), sucrose (*ptsB*, HAPS1008) and mannose (*manXYZ*, HAPS1732–1730). These confirmed genotypes support the previously observed biochemical patterns of sugar fermentation in *H. parasuis* [26].

Complete sets of genes coding for enzymes involved in glycolysis and gluconeogenesis, the tricarboxylic acid (TCA) cycle, as well as the pentose phosphate pathway, were identified in the SH0165 genome (Figure 3). The Entner–Doudoroff pathway, an alternative bypass of classical glycolysis, was not encoded in the *H. parasuis* SH0165 genome, as genes *edd* and *eda* encoding 6-phosphogluconate dehydratase and 2-keto-3-deoxygluconate 6-phosphate aldolase, respectively, are absent. By contrast, *H. somni* 129PT and *H. influenzae* Rd both have an active Entner–Doudoroff pathway responsible for the conversion of gluconate-6-phosphate to pyruvate [11]. In addition, *H. parasuis* SH0165 possessed an enzyme (307 amino acids, aa), encoded by the *rbsK2* (HAPS1688) gene, that was homologous to the fructokinase CscK (307 aa) of *Escherichia coli* EC3132, which can catalyze the phosphorylation of fructose to fructose-6-phosphate [27]. Rbsk2 is capable of metabolizing fructose as an alternative start point to glycolysis. However, *H. influenzae* Rd, *H. somni* 129PT and *H. ducreyi* 35000HP all lack this gene. Five *H. parasuis* genes coding for enzymes involved in the conversion of pyruvate were identified, which are necessary for ATP production through lactic acid fermentation (*ldhA*, HAPS2117; *dld*, HAPS1875), and for the generation of formate (*pflB*, HAPS0155) and acetate (*pta*, HAPS0393; *ackA*, HAPS0392). Besides fermentation, *H. parasuis* may conduct the entire oxidation of glucose derivatives aerobically to release energy through an intact TCA system (Figure 3). In contrast, several other sequenced species in the genus *Haemophilus*, e.g., *H. influenzae*, *H. ducreyi* and *H. somni*, have been found to harbor deficiencies in the TCA cycle, as they are missing one or three genes coding for citrate synthase, aconitase and isocitrate dehydrogenase [11]. In addition, genes *aceA* and *aceB* encoding isocitrate lyase and malate synthase, respectively, which code for key enzymes involved in the glyoxylate shunt, are absent in *H. parasuis* SH0165. Moreover, all known sequenced genomes of *Haemophilus* spp. are lacking the entire glyoxylate bypass.

*H. parasuis* may perform not only aerobic respiration but also anaerobic respiration, as approximately 61 genes involved in the branched electron respiration transport chains were identified in the SH0165 genome (Table S1). Besides cytochrome D ubiquinol oxidase encoded by *cydAB* (HAPS0067, 0068), which is used for aerobic respiration in the presence of oxygen, *H. parasuis* SH0165 possesses an intact *napFDAGHBC* (HAPS1796–1790) operon encoding a putative periplasmic nitrate reductase responsible for the reduction of terminal electron acceptor nitrates in anaerobic environments [28], [29].

To effectively control gene expression in response to environmental stimuli, *H. parasuis* SH0165 encodes about 94 genes related to regulatory functions (Table S2). Genes encoding conserved global regulatory proteins, such as Crp (HAPS2043), CyaA (HAPS0993), Fnr (HAPS0167), ScrR (HAPS1203), Mlc (HAPS0390) and CsrA (HAPS0464), are present and intact in the SH0165 genome. These regulators are able to play a role in activating or repressing the transcription of genes involved in carbon metabolism or other central metabolisms [30], [31]. In addition, three predicted two-component signal transduction systems, encoded by *cpxAR*, *arcAB*, and *qseBC*, were also identified in the genome of *H. parasuis* SH0165. Two-component systems are known to mediate adaptive responses to various environmental signals [32]. For instance, under anaerobic conditions, the ArcA-ArcB system may upregulate genes for anaerobic respiration while downregulate genes for aerobic respiration and fermentation [33], [34].

### NAD biosynthesis

*H. parasuis* requires supplementation of the culture media with NAD (V factor) for growth, but NAD is not necessary for the *in vitro* growth of *H. somni* or *H. ducreyi* [11]. Corresponding to these phenotypic differences, gene *nadV*, which encodes the nicotinamide phosphoribosyltransferase responsible for NAD salvage, is present in the genomes of *H. somni* and *H. ducreyi* (HS0002, HD1447 and HD1455) [11], [35], but is absent from the SH0165 genome. This is the first time that the NAD-dependent growth of *H. parasuis* can be explained genetically [1].

### Heme biosynthesis

Unlike *H. influenzae* and *H. ducreyi*, *H. parasuis* can grow well *in vitro* without requiring additional supplementation of iron porphyrin (X factor) [1]. This is because the heme biosynthetic pathway is present and intact in *H. parasuis* SH0165. An entire set of genes encoding enzymes that are responsible for the conversion of L-glutamate to protoheme were identified in our study (Table S3). Notably, *H. influenzae* Rd and *H. ducreyi* 35000HP were missing the main enzymes necessary for heme biosynthesis, but the animal-originated members within the *Pasteurellaceae* family, e.g. *H. parasuis*, *H. somni*, *A. pleuropneumoniae* and *P. multocida*, all had a complete biosynthetic pathway for the production of the porphyrin ring. Two *hemN* genes (HAPS0238 and 0482) encoding coproporphyrinogen III oxidases (EC: 1.3.99.22) were identified in *H. parasuis* SH0165. Despite the identity between HemN1 and HemN2 only being 23%, they both belong to the *HemN_C* (PF06969) family whose members catalyze the conversion of coproporphyrinogen-III to protoporphyrinogen-IX [36].

Otherwise, the SH0165 genome encodes almost all indispensable enzymes involved in the biosynthesis of amino acids, nucleotides, fatty acids and cofactors. The catalase encoded by *hktE* (HAPS2238) may support a catalase-positive phenotype [2]. It is worth noting that genes *ureABC* encoding urease are all absent from the *H. parasuis* SH0165 genome, explaining the urease-negative characteristic of this species [2].

### Natural competence

Many bacteria are competent, indicating that they have the ability to actively acquire extracelluar DNA from the environment [37]. In comparison with the competence genes in *H. influenzae* and *Mannheimia haemolytica* [38], [39], the *H. parasuis* SH0165 genome also contains homologous genes encoding proteins involved in the assembly of the DNA uptake machinery. Outer membrane proteins ComE (HAPS2289) and PilA (HAPS2013) play a potential role in binding liberated DNA segments at the cell surface [38]. Orthologs of proteins that function in the transport of DNA across the cell membrane, periplasmic space, then into the cytoplasm, include lipoprotein ComL (HAPS0926), periplasmic protein ComF (HAPS1497) and cytoplasmic membrane proftein ComEA (HAPS0844) [38]. Additionally, several cytoplasmic competence proteins coding genes were identified in the *H. parasuis* SH0165 genome, including *comA* (HAPS2285), *dprA* (HAPS1573) and *comM* (HAPS1740) [39]. These genetic components may contribute to the natural competence of *H. parasuis* [40].

**Figure 4:**
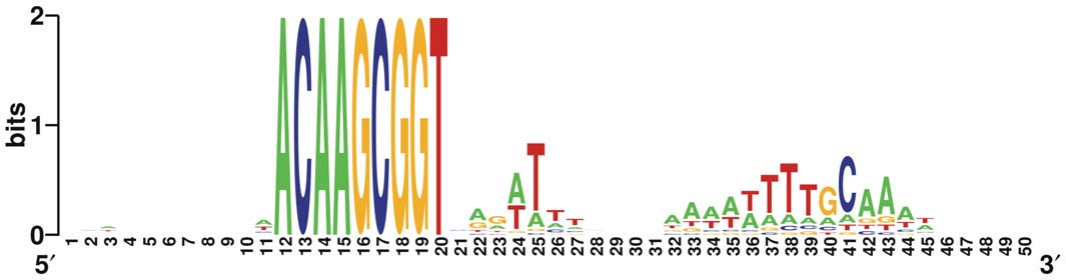
WebLogos from alignments of the sequences flanking the *H. parasuis* uptake signal sequences (USSs) in the complete genome of SH0165. Logos based on 50-bp fragments containing the exact 9-bp USS plus 11 bases upstream and 30 bases downstream.

It has been shown that *Pasteurellaceae* species preferentially take up short DNA sequences, called uptake signal sequences (USS), that are overrepresented in their respective genomes [41]. In *H. influenzae*, the highly redundant USS has the sequence AAGTGCGGT, and this is also the most common uptake sequence in the genomes of *H. somni*, *A. actinomycetemcomitans*, *P. multocida* and *M. succiniciproducens* [41]. However, in the genome sequence of *H. parasuis* SH0165, the most frequent nine-mer repeat is ACAAGCGGT, being present in 523 copies, whereas only 109 copies of the *H. influenzae* core USS were identified in *H. parasuis*. Moreover, the core USS of *H. parasuis* is identical to the most common nine-mer repeats previously identified in *A. pleuropneumoniae* and *M. haemolytica* [41]. Figure 4 shows the 50-bp consensus sequences flanking the core USSs in the *H. parasuis* SH0165 genome. Two AT-rich motifs were observed downstream of the *H. parasuis* USSs and similar patterns of sequence conservation have been found in *M. haemolytica* and *A. pleuropneumoniae*, further confirming that these three microorganisms belong to the same evolutionary subclade [41], [42].

### Adherence and secretion

Fimbriae (namely pili) are a common virulence factor that mediate bacterial adherence to mucosal epithelia [43]. Fimbria-like structures have been observed on the surface of *H. parasuis* after *in vivo* passage [44]. As previously reported, a gene cluster *pilABCD* coding for type IV fimbriae has been identified in a number of Gram-negative pathogens in the genera of *Haemophilus*, *Actinobacillus*, *Pseudomonas*, *Vibrio* and others [45], [46]. *H. parasuis* SH0165 also possesses four type IV fimbrial genes encoding the major structural unit PilA (HAPS2013) and three biogenesis proteins PilBCD (HAPS2011–2009) for mediating bacterial adherence. The proteins encoded by these CDSs share 68%, 68%, 50% and 44% identity with the ApfABCD proteins of *A. pleuropneumoniae* HK361, respectively [46]. In addition to PilABCD, *H. parasuis* SH0165 also encodes a protein (HAPS2143, 22.3 kDa) that shares 54% similarity with *Pseudomonas aeruginosa* PilF (22.4 kDa), which is involved in type IV fimbrial biogenesis and twitching motility [47].

Genes coding for proteins that constitute the classical Sec and Tat secretion systems were identified in the genome of *H. parasuis* SH0165 (Table 3), which are required for protein secretion and trafficking. Ten paralog genes *vtaA* coding for virulence-associated trimeric autotransporters of the type V secretion system were also found in SH0165, the products of which are generally exported through the cytoplasmic membrane *via* the Sec system [48]. Recent studies have indicated that the *H. parasuis vtaA* genes reveal diversity and the expressed products are good candidate immunogens [49], [50]. Furthermore, three potential autotransporters related to the type V protein secretion pathway were encoded in the SH0165 genome, including two EspP proteins (HAPS1378, 771 aa; HAPS1381, 780 aa) and AidA (HAPS0753, 858 aa), all of which have a C-terminal translocator domain (PF03797) responsible for the transport of the N-terminal passenger domain across the outer membrane [51]. The amino acid sequence identity between both EspP proteins was high (73%), indicating that they may be encoded by duplicate copies of the same gene.

**Table 3:** Genes encoding proteins with a putative role in adherence and secretion of strain SH0165.

CDS no.	Name	Function
HAPS0003	*vtaA1*	virulence-associated trimeric autotransporter
HAPS0129	*lepB*	signal peptidase I
HAPS0206	*vtaA2*	virulence-associated trimeric autotransporter
HAPS0226	*secA*	preprotein translocase subunit SecA
HAPS0229	*nlpE*	lipoprotein copper homeostasis and adhesion, NlpE
HAPS0250	*lolA*	outer-membrane lipoprotein carrier protein precursor
HAPS0368	*vtaA3*	virulence-associated trimeric autotransporters
HAPS0427	*secG*	preprotein translocase subunit SecG
HAPS0452	*vtaA4*	virulence-associated trimeric autotransporters
HAPS0499	*vtaA5*	virulence-associated trimeric autotransporters
HAPS0519	*vtaA6*	virulence-associated trimeric autotransporters
HAPS0687	*vtaA7*	virulence-associated trimeric autotransporters
HAPS0753	*aidA*	Type V secretory pathway, adhesin AidA
HAPS0764	*fimB*	fimbrial assembly chaperone
HAPS0956	*vtaA8*	virulence-associated trimeric autotransporters
HAPS0966	*secF*	preprotein translocase subunit SecF
HAPS0967	*secD*	preprotein translocase subunit SecD
HAPS0968	*yajC*	preprotein translocase subunit YajC
HAPS1305	*lspA*	lipoprotein signal peptidase
HAPS1378	*espP1*	extracellular serine protease (autotransporter)
HAPS1381	*espP2*	extracellular serine protease (autotransporter)
HAPS1391	*tatC*	Sec-independent protein translocase protein TatC
HAPS1392	*tatB*	Sec-independent protein translocase protein TatB
HAPS1393	*tatA*	Sec-independent protein translocase protein TatA
HAPS1394	*yidC*	putative inner membrane protein translocase component
HAPS1434	*secY*	preprotein translocase subunit SecY
HAPS1477	*secB*	preprotein translocase subunit SecB
HAPS1508	*ffh*	signal recognition particle GTPase
HAPS1511	*vtaA9*	virulence-associated trimeric autotransporters
HAPS1817	*secE*	preprotein translocase subunit SecE
HAPS1856	*ftsY*	cell division protein, signal recognition particle GTPase
HAPS2009	*pilD*	Tfp pilus assembly pathway, fimbrial leader peptidase PilD
HAPS2010	*pilC*	Tfp pilus assembly pathway, component PilC
HAPS2011	*pilB*	Tfp pilus assembly pathway, ATPase PilB
HAPS2013	*pilA*	Tfp pilus assembly protein, major pilin PilA
HAPS2037	*lolB*	outer membrane lipoprotein LolB
HAPS2063	*vtaA10*	virulence-associated trimeric autotransporters
HAPS2143	*pilF*	fimbrial biogenesis and twitching motility protein
HAPS2243	*pulG*	Type II secretory pathway, pseudopilin PulG

### Biosynthesis of surface polysaccharide

LPSs are the primary structural and functional components of the Gram-negative bacterial outer membrane, which can be recognized and targeted by the mammalian immune system [52]. Typical LPS molecules consist of three covalently linked biochemical moieties: the lipid A, the core oligosaccharide and the major surface-exposed O-specific antigen (known as the O polysaccharide). The O polysaccharide is composed of many repeats of an oligosaccharide unit (O unit) and plays a vital role in bacterial adherence, invasion and immune evasion [53]. The LPSs of *H. parasuis*, also termed lipo-oligosaccharides (LOSs) in this bacterium, are considered a virulence factor associated with disseminated intravascular coagulation and thrombosis [8], [54].

**Figure 5:**
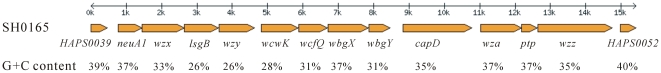
The genetic organization of the LPS O-antigen biosynthetic region in *H. parasuis* SH0165. The coding sequences (CDSs) are drawn to scale, with 1 kb increments indicated.

All genes coding for enzymes involved in the biosynthesis of LOS were identified in the SH0165 genome (Table S4). Unsurprisingly, according to sequence comparisons and function assignment, genes essential for the synthesis of lipid A (*lpxC*, *kdsA*, *lpxB*, *kdsB*, *lpxH*, *lpxK*, *lpxD*, *lpxA*, *kdtA*, *lpxM*, *kdsC* and *lpxL*) and core oligosaccharide (*rfaE*, *rfaF*, *rfaD*, *lgtF* and *gmhA*) are present and highly conserved among the genus *Haemophilus*. Furthermore, a 13,964 bp genomic region that is likely associated with O-antigen biogenesis was predicted in the *H. parasuis* SH0165 genome. Twelve putative CDSs were identified within this potential O-antigen region located between HAPS0039 and HAPS0052, and all of these share the same transcriptional orientation (Figure 5). Like many other Gram-negative bacteria, the *H. parasuis* O-antigen gene cluster had a G+C content (32.8%) lower than that of the SH0165 genome (40.0%). Nine of these CDSs could be divided into three categories according to their functional roles in the biogenesis of O-antigen [55]: sugar synthesis-related genes (*neuA1* and *wbgX*); glycosyltransferase genes (*lsgB*, *wcwK*, *wcfQ* and *wbgY*); and O unit processing genes *wzx*, *wzy* and *wzz* involved in the assembly of O-polysaccharide.

**Figure 6:**
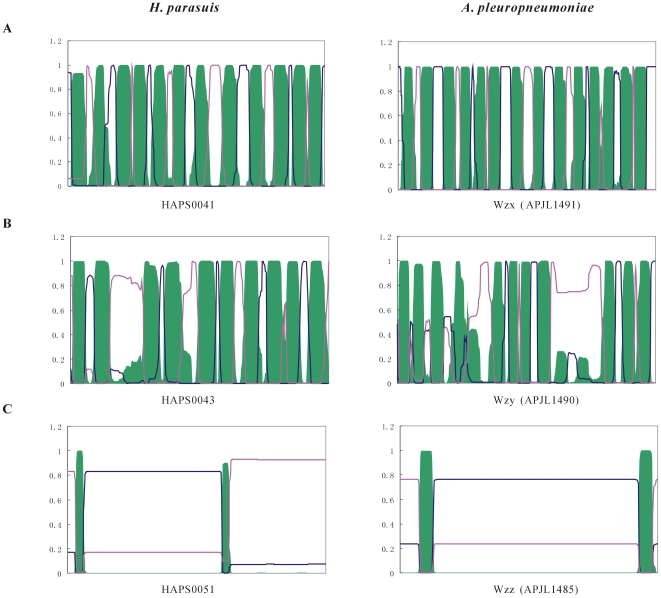
Schematic comparisons of the topological models of transmembrane proteins and functional assignment of genes encoding enzymes with a putative role in the biogenesis of O-antigen. A. HAPS0041 (APJL1491, *wzx*), encoding an O-antigen flippase; B. HAPS0043 (APJL1490, *wzy*), encoding a putative O-antigen polymerase; C. HAPS0051 (APJL1485, *wzz*), encoding an O-antigen chain length determining protein. All the amino acid sequences were retrieved from the genomes of *H. parasuis* SH0165 and *A. pleuropneumoniae* JL03. The posterior probability of the transmembrane helix, intracellular side and extracellular side are indicated in green, red and blue, respectively.

In general, O-unit-processing enzymes (Wzx, Wzy and Wzz) in diverse Gram-negative bacteria show considerable sequence variation but share conserved topologies in membrane spanning regions [53]. The *wzx* (HAPS0041) gene of *H. parasuis* encodes an O-antigen flippase (398 aa) which has 12 predicted transmembrane helices and shares 48% identity and 69% similarity with a Wzx homologue (404 aa) from the *Shigella boydii* O-antigen gene cluster [56]. Although BLASTP searches did not detect any significant homologues of the product of HAPS0043, it was predicted to be an inner-membrane protein with 10 transmembrane helices that contains a large periplasmic loop of 44 amino acid residues (Figure 6), a topology typical of all known O-antigen polymerases [57], [58]. Thus, we propose that HAPS0043 of *H. parasuis* is the O-antigen polymerase gene, *wzy*. More experimental works are still required to verify the function of *H. parasuis* Wzy in the O-antigen-processing process.

As is well known, sialylated oligosaccharide units in bacterial polysaccharides play a role in evading the host immune response by mimicking the glycolipid components of mammalian tissues [59]. A CMP-NeuNAc synthetase (N-acetylneuraminic acid cytidylsynthetase) (226 aa) encoded by the *neuA1* (HAPS0040) gene of *H. parasuis* SH0165 shares 63% identity and 81% similarity with NeuA (229 aa) of *H. ducreyi* 35000 [60]. Furthermore, LsgB (322 aa), encoded by HAPS0042 of *H. parasuis*, exhibits about 51% similarity and 31% identity with Lst (371 aa) of *Neisseria meningitidis* and has a glycosyltransferase family 52 domain (PF07922) bearing alpha-2,3-sialytransferase activity. Lst encodes a CMP-NeuNAc transferase that is responsible for the linkage α-NeuNAc-(2–3)-Gal [61]. NeuA and Lst have been reported to be essential for the sialic acid-containing glycoform of *H. ducreyi* LOS [62]. Therefore, the identification of these two homologues in *H. parasuis* may indicate that this organism can synthesize a sialylated LPS glycoform. In addition, a putative nucleotide sugar aminotransferase WbgX encoded by HAPS0046 exhibits 85% similarity and 72% identity to the *Shigella sonnei* homologue that is involved in the synthesis of 2-acetamino-4-amino-2,4,6-trideoxy-D-galactose (4n-FucNAc) [63].

The *H. parasuis* SH0165 O-antigen gene cluster encodes four glycosyltransferases that sequentially add diverse sugar residues to make an expected four-sugar repeating O-unit. Besides LsgB, WcfQ (272 aa) encoded by HAPS0045 belongs to the glycosyl transferase family 2 (PF00535, E_value = 2×10^−18^) whose members can transfer different sugar donors to receptors. *H. parasuis* WcfQ is also 58% similar to the putative glycosyltransferase WcfQ (268 aa) involved in the type A capsular polysaccharide biosynthesis of *Bacteroides fragilis* 9343 [64]. Gene *wcwK* (HAPS0044) encodes an enzyme (343 aa) that shares 35% identity (53% similarity) with a capsular polysaccharide phosphotransferase (335 aa) found in *Streptococcus pneumoniae* [65]. WbgY encoded by HAPS0047 has 79% similarity and 59% identity to a bacterial sugar transferase involved in the first step of *Plesiomonas shigelloides* O17 O-antigen synthesis [63].

The deduced protein products (CapD, Wza, Ptp and Wzz) of the last four genes (HAPS0048–0051) in the *H. parasuis* SH0165 O-antigen chain cluster are homologous to the proteins WbfY, Wza, Wzb and Wzc encoded in the *Vibrio vulnificus* group 1 capsular polysaccharide operon, sharing 52%, 53%, 57% and 50% aa identity, respectively [66]. It has recently been shown that CapD and WbgY are also virulence-associated genes of *H. parasuis* through the suppression subtractive hybridization of a highly virulent and a nonvirulent strain [67].

Another gene cluster involved in LOS biosynthesis was identified in the *H. parasuis* SH0165 genome, which also has a low G+C content (28.4%), indicating that this DNA region may originate from a species other than *H. parasuis*. This cluster contains six putative sugar transferase genes, *lbgB*, *rfaF2*, *lbgA*, *rfaG*, *wabH* and *lpsA*, which appear to be unique to *H. parasuis*, with most of them being absent from other species within the genus *Haemophilus* (Table S4). The products encoded by these CDSs belong to the glycosyltransferase family 1 (HAPS1021, 1022), 9 (HAPS1017, 1019) and 25 (HAPS1020, 1023), respectively.

Interestingly, nine genes were identified in the *H. parasuis* SH0165 genome that encode enzymes homologous to those encoded by the *wec* genes associated with the synthesis of Enterobacterial Common Antigen (ECA), a complex cell surface glycolipid found in all Gram-negative enteric bacteria [68]. Unlike the *wec* gene cluster in the *E. coli* K12 chromosome [69], the *H. parasuis* ECA-like related genes, *wecA*, *wecB*, *wecC*, *rmlB*, *wecD*, *wecE*, *wecF*, *wzyE* and *wecG*, are dispersed throughout the SH0165 chromosome (Table S4). The identification of these genes provides genetic evidence that *H. parasuis* may produce a putative ECA-like glycoconjugate. It is worth mentioning that the *wec* homologues are also present and linked in a cluster in *H. ducreyi* 35000HP [70].

### Virulence-related genes

A number of enzymes, including neuraminidase and proteases, may be important for the virulence of *H. parasuis*. The gene *nanH* (HAPS1616) coding for a potential virulence factor, neuraminidase (sialidase), was identified in the SH0165 genome and its product may function in *H. parasuis* colonization [71]. The *H. parasuis* SH0165 genome contains approximately 37 genes encoding different proteases that may contribute to virulence (Table S5). The majority of protease-coding genes are conserved among members of the *Pasteurellaceae*, perhaps due to their role in proteolysis for protein quality control, but several other proteases (encoded by HAPS0648, 1928 and 2032) may be unique to *H. parasuis*. A *clpPX* (HAPS2006, 2007) operon encodes the structural subunits of the ATP-dependent Clp protease which proteolysis-function has been reported to be pivotal to the secretion processes of Gram-negative pathogens [72]. The gene *lon* (HAPS1011) encodes a cytoplasmic serine protease (800 aa) that shares 70% identity with *Salmonella enterica* serovar Typhimurium Lon protease (784 aa), a potential target of antimicrobial therapy [73].

### Iron acquisition and utilization

Iron plays a crucial role in the basic physiological functions of mammalian pathogens, e.g., ATP synthesis, formation of heme, bacterial survival and persistent infection. To overcome iron restriction in host environments, many pathogenic bacteria have evolved different approaches for iron uptake, including synthesizing certain iron-chelating compounds, called siderophores, or scavenging host iron-binding complexes [74]. Unlike other bacterial pathogens that can produce high-affinity siderophores, such as enterobactin, alcaligin and pyochelin, it seems that *H. parasuis* may be deficient in production of these molecules, as reflected by the lack of related biosynthetic genes [74]. However, *H. parasuis* SH0165 was found to encode at least 37 genes involved in the capture and utilization of iron from porcine transferrin, heme and heme–hemopexin (Table S6).

negative bacteria [75]. Two clusters of consecutive genes *exbB1*–*exbD1*–*tonB1* (257 aa) (HAPS1366–1364) and *tonB2* (267 aa)–*exbB2*–*exbD2* (HAPS2220–2222) were identified in the SH0165 genome, which are responsible for the formation of the TonB1 and TonB2 systems, respectively. Gene components of the TonB2 system have been described previously in *H. parasuis* [9]. Although the periplasmic proteins TonB1 and TonB2 share low sequence identity (24%), they contain an identical domain (PF03544) involved in the transfer of energy to transferrin binding proteins (TbpB and TbpA), both homologues of which are responsible for transport of iron across the outer membrane of *A. pleuropneumoniae* [76]. *H. parasuis* TbpA and TbpB (HAPS2224, 2223) have low sequence identity (28% and 42%) with *H. influenzae* Tbp1 and Tbp2 (HI0994, 0995), respectively, but are highly homologous to the *A. pleuropneumoniae* TbpA and TbpB (APJL1597, 1598) proteins, sharing 93% and 87% identity, respectively [77], [78]. Consequently, the divergent residues inferred from the sequence alignments may help determine why *H. parasuis* and *A. pleuropneumoniae* preferentially bind porcine transferrin but not human transferrin [79].

Besides the TonB-dependent systems, *H. parasuis* seems to have various ABC transport systems involved in the uptake of extracellular iron, hemin and hemoprotein. A hemin transport system encoded by the *hmuTUV* operon (HAPS1572–1570) was identified in the SH0165 genome and was found to be homologous to a family of ABC transporters (HemTUV, ShuTUV) that function in transporting free heme into the cytoplasm [80]. Orthologs of *yfeABCD* (HAPS1097–1100) and *fbpABC* (HAPS1129–1127) were found in *A. actinomycetemcomitans* and *M. haemolytica*, respectively, and their protein products make up periplasmic ABC transport complexes involved in iron acquisition [81], [82]. In *H. parasuis*, the periplasmic iron transport system FbpABC together with the transferrin outer membrane receptor complex TbpAB may form another iron uptake pathway, which is used by *Neisseria* species to transport ferric iron into the cell [83].

Although the intact *fhuCDBA* operon involved in ferric hydroxamate uptake has been reported in strain Nagasaki of *H. parasuis* serovar 5 [84], it was found to be truncated in the SH0165 genome, only consisting of the 5′ portion of *fhuC'* (HAPS0095) and 3′ portion of *fhuA'* (HAPS0096), and lacking *fhuDB*. Genes *hhdB'* and *hhdA'* (HAPS1445, 1446), encoding two components of a putative hemolysin export system, and *cirA'* (HAPS0477), encoding an iron transporter, are also likely to be non-functional due to frameshift mutations [85].

In this study, we reported the complete genome sequence of *H. parasuis* strain SH0165, a highly virulent strain isolated in China, and further performed a comparative genomic analysis. Genomic and phylogenetic comparisons indicated that *H. parasuis* and *A. pleuropneumoniae* may derive from a recent common ancestor. Together with a full set of the relevant metabolic genes, we confirmed that *H. parasuis* generates ATP *via* both fermentation and respiration with an intact TCA cycle for anabolism. Furthermore, genes and operons related to bacterial virulence factors, such as surface polysaccharides, fimbriae and iron acquisition systems, were identified at the genomic level and compared with known virulence genes from other pathogens. A putative Wzy/Wzx-dependent biosynthetic pathway for O-chain polysaccharide was proposed and requires further experimental confirmation in *H. parasuis*. Our findings should provide a genetic foundation for future research into the mechanisms of pathogenesis of *H. parasuis* and will accelerate the development of safe and effective vaccines to prevent and control this severe swine disease.

## Supporting Information

Table S1 **Genes encoding enzymes involved in central metabolism of** ***H. parasuis*** **SH0165 and orthologs present in genomes of five representative species within** ***Pasteurellaceae*** **.**
(XLS)

Table S2 **Genes coding for proteins related to regulatory functions of strain SH0165.**
(XLS)

Table S3 **Heme biosynthetic enzymes encoded in the genome of** ***H. parasuis*** **SH0165 and orthologs from five representative** ***Pasteurellaceae*** **genomes.**
(DOC)

Table S4 **Genes encoding enzymes with a role in surface polysaccharides of strain SH0165 and orthologs present in genomes of three representative** ***Haemophilus*** **spp.**
(DOC)

Table S5 **Putative protease-encoding genes in the** ***H. parasuis*** **SH0165 genome.**
(XLS)

Table S6 **Genes encoding proteins involved in iron metabolism of** ***H. parasuis*** **SH0165 and orthologous proteins from five representative genomes within** ***Pasteurellaceae*** **.**
(DOC)
