# Supplementary material for: Genomic Characterization of Haemophilus parasuis SH0165, a Highly Virulent Strain of Serovar 5 Prevalent in China
Source: PLoS One. 2011 May 17;6(5):e19631. doi: 10.1371/journal.pone.0019631 (PMC3096633; doi:10.1371/journal.pone.0019631)
Supplement: Table S3 — Heme biosynthetic enzymes encoded in the genome of H. parasuis SH0165 and orthologs from five representative Pasteurellaceae genomes. (DOC) [file pone.0019631.s003.doc]

**Table S3. Heme biosynthetic enzymes encoded in the genome of *H. parasuis* SH0165 and orthologs from five representative *Pasteurellaceae* genomes**

| Name | Function | *H. parasuis* | *H. somni* | *H. influenzae* | *H. ducreyi* | *A. pleuropneumoniae* | *P. multocida* |
| --- | --- | --- | --- | --- | --- | --- | --- |
| *hemN1* | coproporphyrinogen III oxidase | HAPS0238 | HS0124 | HI0463 | HD1668 | APJL0858 | PM1669 |
| *hemN2* | coproporphyrinogen III oxidase | HAPS0482 | HS0472 | － | － | APJL0982 | PM1833 |
| *hemA* | glutamyl-tRNA reductase | HAPS0733 | HS0810 | － | － | APJL0425 | PM0684 |
| *hemE* | uroporphyrinogen-III decarboxylase | HAPS0795 | HS1540 | － | － | APJL0113 | PM1734 |
| *hemX* | uroporphyrin-III C-methyltransferase | HAPS1004 | HS0046 | － | HD1741 | APJL1026 | PM1814 |
| *hemD* | uroporphyrinogen-III synthase | HAPS1005 | HS0045 | － | － | APJL1027 | PM1813 |
| *hemC* | porphobilinogen deaminase | HAPS1006 | HS0044 | － | － | APJL1028 | PM1812 |
| *hemB* | delta-aminolevulinic acid dehydratase | HAPS1171 | HS0548 | － | － | APJL2036 | PM1692 |
| *hemH* | protoheme ferro-lyase | HAPS1733 | HS1047 | HI1160 | － | APJL1983 | PM0789 |
| *hemL* | glutamate-1-semialdehyde aminotransferase | HAPS1765 | HS1229 | － | － | APJL1583 | PM0462 |
| *hemG* | protoporphyrinogen oxidase | HAPS1976 | HS1569 | － | － | APJL1624 | PM1499 |
| *gltX* | glutamyl-tRNA synthetase | HAPS2152 | HS0456 | HI0274 | HD0320 | APJL1286 | PM1115 |
