# Supplementary material for: Genomic Characterization of Haemophilus parasuis SH0165, a Highly Virulent Strain of Serovar 5 Prevalent in China
Source: PLoS One. 2011 May 17;6(5):e19631. doi: 10.1371/journal.pone.0019631 (PMC3096633; doi:10.1371/journal.pone.0019631)
Supplement: Table S4 — Genes encoding enzymes with a role in surface polysaccharides of strain SH0165 and orthologs present in genomes of three representative Haemophilus spp. (DOC) [file pone.0019631.s004.doc]

**Table S4. Genes encoding enzymes with a role in surface polysaccharides of strain SH0165 and orthologs present in genomes of three representative *Haemophilus* spp**

| *H. parasuis* | Name | Putative function | *H. somni* | *H. influenzae* | *H. ducreyi* |
| --- | --- | --- | --- | --- | --- |
| HAPS0040 | *neuA1* | CMP-N-acetylneuraminic acid synthetase | HS0706 | HI1279 | HD0685 |
| HAPS0041 | *wzx* | O-antigen flippase | － | HI0867 | HD0882 |
| HAPS0042 | *lsgB* | Beta-galactoside alpha-2,3-sialyltransferase | － | HI1699 | － |
| HAPS0043 | *wzy* | putative O antigen polymerase | － | － | － |
| HAPS0044 | *wcwK* | glycosyltransferase (stealth protein wcwK) | － | － | － |
| HAPS0045 | *wcfQ* | extracellular polysaccharide glycosyltransferase | － | HI1695 | HD0886 |
| HAPS0046 | *wbgX* | DegT/DnrJ/EryC1/StrS aminotransferase | － | － | HD1840 |
| HAPS0047 | *wbgY* | putative glycosyltransferase | － | － | － |
| HAPS0048 | *capD* | polysaccharide biosynthesis protein CapD | － | － | － |
| HAPS0049 | *wza* | polysaccharide export protein, periplasmic protein | － | － | － |
| HAPS0050 | *ptp* | cytoplasmic tyrosine phosphatase | － | － | － |
| HAPS0051 | *wzz* | protein-tyrosine kinase, regulator of length of O-antigen component of lipopolysaccharide chains | － | － | － |
| HAPS0125 | *lpxC* | UDP-3-O-[3-hydroxymyristoyl] N-acetylglucosamine deacetylase | HS0364 | HI1144 | HD0816 |
| HAPS0137 | *gmhA1* | phosphoheptose isomerase | HS1238 | HI1181 | HD1228 |
| HAPS0295 | *-* | exopolysaccharide biosynthesis protein | － | HI1244 | － |
| HAPS0361 | *gmhB* | D,D-heptose 1,7-bisphosphate phosphatase | HS1532 | HI0621.1 | HD1666 |
| HAPS0735 | *rfaE* | bifunctional heptose 7-phosphate kinase, heptose 1-phosphate adenyltransferase | HS0576 | HI1526 | HD1182 |
| HAPS0747 | *gmhA2* | putative phosphoheptose isomerase | HS0738 | HI1657 | HD0801 |
| HAPS0787 | *kdsA* | 2-dehydro-3-deoxyphosphooctonate aldolase | HS0946 | HI1557 | HD0857 |
| HAPS0847 | *rfaF1* | ADP-heptose:LPS heptosyltransferase II | HS1612 | HI1105 | HD0653 |
| HAPS0849 | *manB* | phosphomannomutase | HS1670 | HI0740 | HD1507 |
| HAPS0965 | *lpxB* | lipid-A-disaccharide synthase | HS1358 | HI1060 | HD0846 |
| HAPS0972 | *kdsB* | 3-deoxy-manno-octulosonate cytidylyltransferase | HS0658 | HI0058 | HD0334 |
| HAPS0978 | *rmlB* | dTDP-glucose 4,6-dehydratase | HS0707 | HI0873 | HD0687 |
| HAPS0984 | *lgtF* | UDP-glucose--lipooligosaccharide glucosyltransferase | HS0291 | HI0653 | HD1201 |
| HAPS1017 | *lbgB* | D-glycero-D-manno-heptosyltransferase | － | － | HD1720 |
| HAPS1019 | *rfaF2* | ADP-heptose:LPS heptosyltransferase | － | － | － |
| HAPS1020 | *lbgA* | lipooligosaccharide galactosyltransferase I | － | － | HD1721 |
| HAPS1021 | *rfaG* | glycosyl transferase, group 1 | － | － | － |
| HAPS1022 | *wabH* | glycosyl transferase, group 1 | － | － | － |
| HAPS1023 | *wzyE* | lipooligosaccharide biosynthesis protein | － | HI0765 | HD0472 |
| HAPS1031 | *lpxH* | UDP-2,3-diacylglucosamine hydrolase | HS0498 | HI0735 | HD1938 |
| HAPS1032 | *vimF* | glycosyltransferases | － | － | － |
| HAPS1038 | *galE* | UDP-glucose-4-epimerase | HS0789 | HI0351 | HD0829 |
| HAPS1054 | *lpxK* | tetraacyldisaccharide 4'-kinase | HS0656 | HI0059 | HD0217 |
| HAPS1059 | *wecG* | UDP-N-acetyl-D-mannosaminuronic acid transferase, teichoic acid biosynthesis protein | － | － | HD1833 |
| HAPS1060 | *wzyE* | putative enterobacterial common antigen polymerase | － | － | HD1835 |
| HAPS1061 | *wecF* | 4-alpha-L-fucosyltransferase | － | － | HD1836 |
| HAPS1268 | *lpxD* | UDP-3-O-[3-hydroxymyristoyl] glucosamine N-acyltransferase | HS0978 | HI0915 | HD1189 |
| HAPS1270 | *lpxA* | UDP-N-acetylglucosamine acyltransferase | HS1359 | HI1061 | HD1187 |
| HAPS1412 | *coaD* | phosphopantetheine adenylyltransferase | HS1589 | HI0651 | HD0453 |
| HAPS1413 | *kdtA* | 3-deoxy-D-manno-octulosonic-acid transferase | HS1590 | HI0652 | HD0454 |
| HAPS1575 | *rfaD* | ADP-L-glycero-D-mannoheptose-6-epimerase | HS1613 | HI1114 | HD1890 |
| HAPS1668 | *lpxM* | lipid A biosynthesis (KDO)2-(lauroyl)-lipid IVA acyltransferase | HS1202 | HI0199 | HD0404 |
| HAPS1766 | *wecA* | undecaprenyl-phosphate alpha-N-acetylglucosaminyltransferase | － | HI1716 | HD1844 |
| HAPS1768 | *wecB* | UDP-N-acetylglucosamine 2-epimerase | － | － | HD1843 |
| HAPS1769 | *wecC* | UDP-N-acetyl-D-mannosamine dehydrogenase | － | － | HD1842 |
| HAPS2142 | *kpsF* | arabinose-5-phosphate isomerase | HS0918 | HI1678 | HD1168 |
| HAPS2163 | *wecD* | putative TDP-D-fucosamine acetyltransferase | － | － | HD1841 |
| HAPS2164 | *wecE* | TDP-4-keto-6-deoxy-D-glucose transaminase | － | － | HD1840 |
| HAPS2200 | *kdsC* | 3-deoxy-D-manno-octulosonate 8-phosphate phosphatase | HS0919 | HI1679 | HD0297 |
| HAPS2206 | *neuA3* | acylneuraminate cytidylyltransferase/CMP-N-acetylneuraminic acid synthetase | HS0706 | HI1279 | HD0685 |
| HAPS2208 | *lsgE* | putative lipooligosaccharide galactosyltransferase | － | HI1696 | HD0885 |
| HAPS2209 | *lsgF* | UDP-galactose--lipooligosaccharide galactosyltransferase | － | HI1695 | HD0886 |
| HAPS2227 | *lpxL* | lipid A biosynthesis lauroyl acyltransferase | HS0575 | HI1527 | HD1106 |
