# Supplementary material for: Genomic Characterization of Haemophilus parasuis SH0165, a Highly Virulent Strain of Serovar 5 Prevalent in China
Source: PLoS One. 2011 May 17;6(5):e19631. doi: 10.1371/journal.pone.0019631 (PMC3096633; doi:10.1371/journal.pone.0019631)
Supplement: Table S6 — Genes encoding proteins involved in iron metabolism of H. parasuis SH0165 and orthologous proteins from five representative genomes within Pasteurellaceae . (DOC) [file pone.0019631.s006.doc]

**Table S6. Genes encoding proteins involved in iron metabolism of *H. parasuis* SH0165 and orthologous proteins from five representative genomes within *Pasteurellaceae***

| *H. parasuis* | Name | Function | *H. somni* | *H. influenzae* | *H. ducreyi* | *A. pleuropneumoniae* | *P. multocida* |
| --- | --- | --- | --- | --- | --- | --- | --- |
| HAPS0013 | *cyaY* | frataxin-like protein implicated in iron transport | HS1712 | HI0727.1 | － | APJL1136 | PM1425 |
| HAPS0092 | *hbpA* | heme-binding protein A precursor | HS0594 | HI0853 | HD0215 | APJL0866 | PM0592 |
| HAPS0094 | *fhuF* | ferric iron reductase involved in ferric hydroximate transport | － | － | － | － | － |
| HAPS0166 | *afuA* | iron (Fe3+) ABC superfamily ATP binding cassette transporter, binding protein | HS0641 | HI0131 | － | APJL0552 | PM0203 |
| HAPS0197 | *afuB* | ABC-type Fe3+ transport system, permease component | HS0640 | HI0129 | － | APJL1417 | PM0956 |
| HAPS0198 | *afuC* | ferric transporter ATP-binding subunit | HS0639 | HI0126 | HD0572 | APJL1418 | PM0957 |
| HAPS0355 | *irp* | iron-regulated outer membrane protein/TonB-dependent receptor | HS0069 | － | － | APJL0930 | PM1428 |
| HAPS1055 | *hugX* | heme utilization protein HuvX | HS0723 | － | HD0037 | APJL1549 | PM0298 |
| HAPS1056 | *hugZ* | heme utilization protein | HS0722 | HI0854 | HD0620 | APJL1066 | PM0299 |
| HAPS1097 | *yfeA* | chelated iron ABC transporter, periplasmic-binding protein | HS0328 | HI0362 | HD1816 | － | PM0400 |
| HAPS1098 | *yfeB* | chelated iron ABC transporter, ATP-binding protein | HS0327 | HI0361 | HD1817 | APJL0280 | PM0399 |
| HAPS1099 | *yfeC* | chelated iron ABC transporter, permease | HS0326 | HI0360 | HD1025 | APJL0129 | PM0398 |
| HAPS1100 | *yfeD* | chelated iron ABC transporter, permease | HS0325 | HI0359 | HD1024 | APJL0128 | PM0397 |
| HAPS1127 | *fbpC* | iron(III) ABC transporter, ATP-binding protein | HS0781 | HI0099 | HD0572 | APJL0290 | PM0049 |
| HAPS1128 | *fbpB* | iron-uptake permease inner membrane protein | HS0782 | － | － | － | PM0050 |
| HAPS1129 | *fbpA* | periplasmic iron-binding protein | HS0783 | － | － | － | PM0051 |
| HAPS1202 | *hbpA* | heme-binding protein A | HS0594 | HI0853 | HD0215 | APJL2060 | PM0592 |
| HAPS1364 | *tonB1* | TobB energy transducing protein | － | － | HD0327 | APJL0076 | － |
| HAPS1365 | *exbD1* | TonB system transport protein ExbD type-2/biopolymer transport protein | HS0690 | HI0252 | HD0328 | APJL0077 | PM1187 |
| HAPS1366 | *exbB1* | biopolymer transport protein ExbB | HS0691 | HI0253 | HD0329 | APJL0078 | PM1186 |
| HAPS1569 | *cjrA* | putative iron-regulated lipoprotein | － | － | － | － | － |
| HAPS1570 | *hmuV* | hemin transport system ATP-binding protein HmuV | － | HI1272 | HD1817 | － | PM1080 |
| HAPS1571 | *hmuU* | hemin transport system permease protein HmuU | － | HI1471 | － | APJL1828 | PM1308 |
| HAPS1572 | *hmuT* | hemin transport system periplasmic protein HmuT | － | － | － | － | － |
| HAPS2126 | *ccmA* | Cytochrome c biogenesis ATP-binding export protein ccmA/heme exporter protein A | HS0393 | HI1089 | HD0786 | APJL1390 | PM0005 |
| HAPS2127 | *ccmB* | cytochrome c-type biogenesis protein/heme exporter protein B | HS0394 | HI1090 | HD0787 | APJL1389 | PM0006 |
| HAPS2128 | *ccmC* | cytochrome c-type biogenesis protein/heme exporter protein C | HS0395 | HI1091 | HD0789 | APJL1388 | PM0007 |
| HAPS2129 | *ccmD* | cytochrome c-type biogenesis protein/heme exporter protein D | － | HI1092 | HD0790 | APJL1387 | PM0008 |
| HAPS2179 | *hxuC* | heme/hemopexin utilization protein C/outer membrane receptor protein, mostly Fe transport | － | HI0262 | － | － | － |
| HAPS2180 | *hxuB* | heme/hemopexin-binding protein B, hemolysin activation/secretion protein | － | HI0263 | － | － | － |
| HAPS2181 | *hxuA* | heme/hemopexin-binding protein A (Heme:hemopexin utilization protein A) | － | HI0264 | － | － | － |
| HAPS2220 | *tonB2* | periplasmic protein, links inner and outer membranes | － | － | － | APJL1601 | － |
| HAPS2221 | *exbB2* | biopolymer transport ExbB protein | HS0259 | HI0385 | HD1777 | APJL1600 | PM0970 |
| HAPS2222 | *exbD2* | biopolymer transport ExbD protein | － | － | － | APJL1599 | － |
| HAPS2223 | *tbpB* | transferrin-binding protein 2 precursor | HS0448 | HI0995 | － | APJL1598 | － |
| HAPS2224 | *tbpA* | transferrin-binding protein 1 precursor | HS0449 | HI0994 | － | APJL1597 | － |
| HAPS2260 | *fur* | ferric uptake regulation protein | HS1211 | HI0190 | HD0367 | APJL1231 | PM0352 |
